# Supplementary material for: Can Circulating Cardiac Biomarkers Be Helpful in the Assessment of LMNA Mutation Carriers?
Source: J Clin Med. 2020 May 12;9(5):1443. doi: 10.3390/jcm9051443 (PMC7290314; doi:10.3390/jcm9051443)
Supplement: Supplementary file 1 [file jcm-09-01443-s001.pdf]

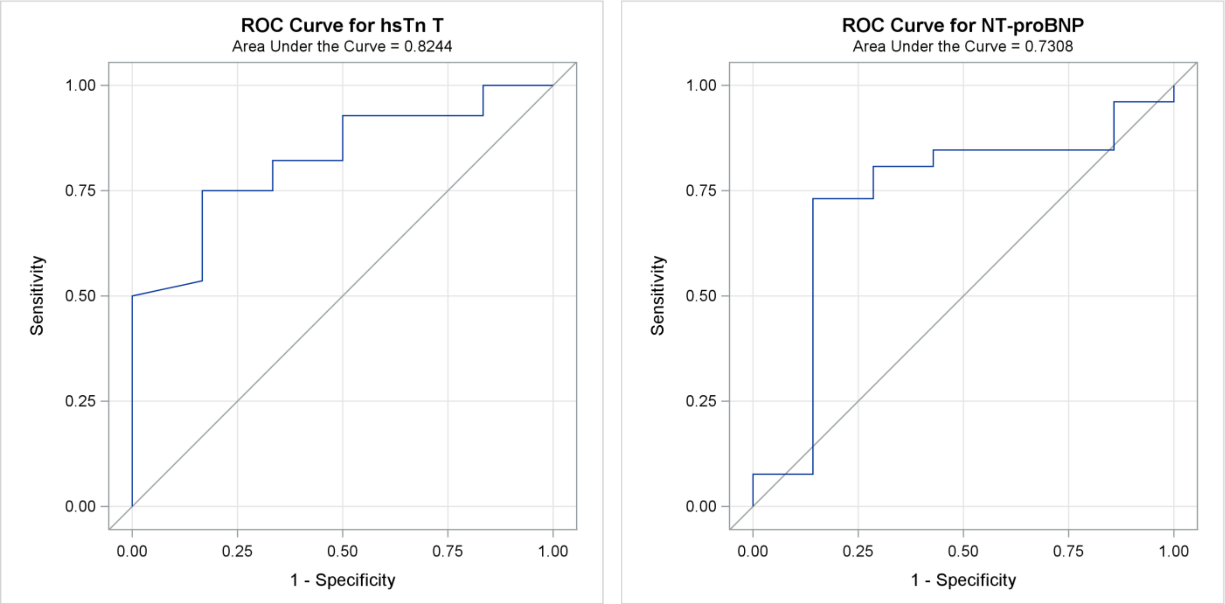

Potential Cut-off Points for hs TnT in MVA Risk Assessment	Sensitivity	Specificity	Sensitivity + Specificity	P
15 ng/l	83.3%	60.7%	144.0%	0.078
20 ng/l	83.3%	75.0%	158.3%	0.014
25 ng/l	66.7%	82.1%	148.8%	0.031

(**a**)

Potential Cut-off Points for NT-proBNP in MVA Risk Assessment	Sensitivity	Specificity	Sensitivity + Specificity	P
125 pg/ml	85.7	69.2	154.9	0.026
150 pg/ml	85.7	73.1	158.8	0.008
200 pg/ml	71.4	76.9	148.3	0.027

(**b**)

Figure S1. Receiver-operating characteristic curves used to assess the cutoff points of the biomarkers for prediction of MVA: (**a**) for hs TnT and (**b**) for NT-proBNP. Legend: hs TnT: highly sensitive Troponin T; MVA: malignant ventricular arrhythmia; NT-proBNP: N-terminal prohormone brain natriuretic peptide; ROC: receiver-operating characteristic.

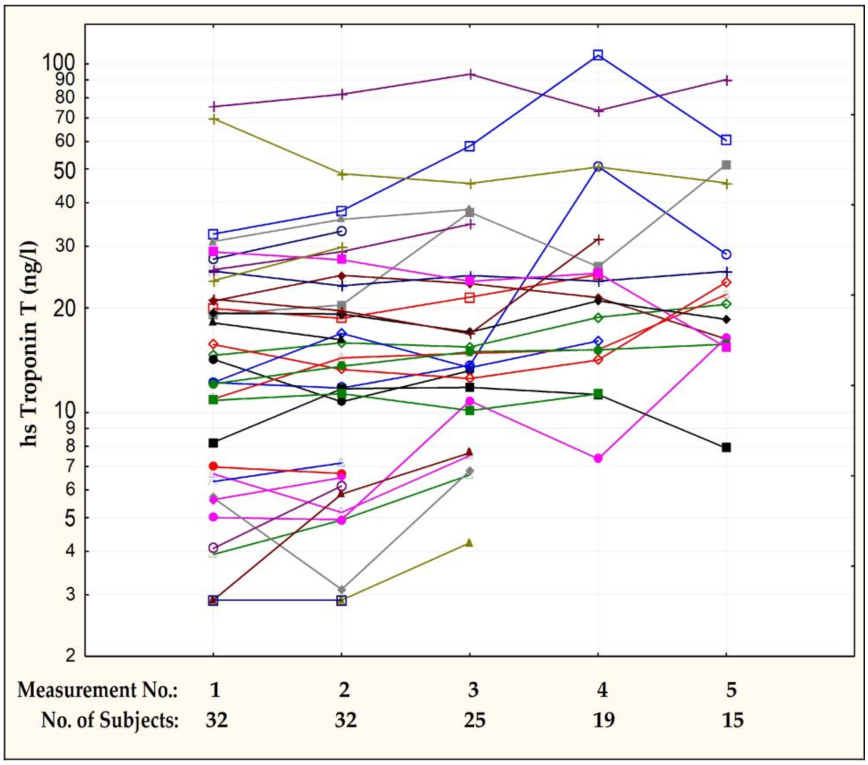

Figure S2. Highly sensitive troponin T level profiles at baseline and during follow-up visits on a logarithmic scale.

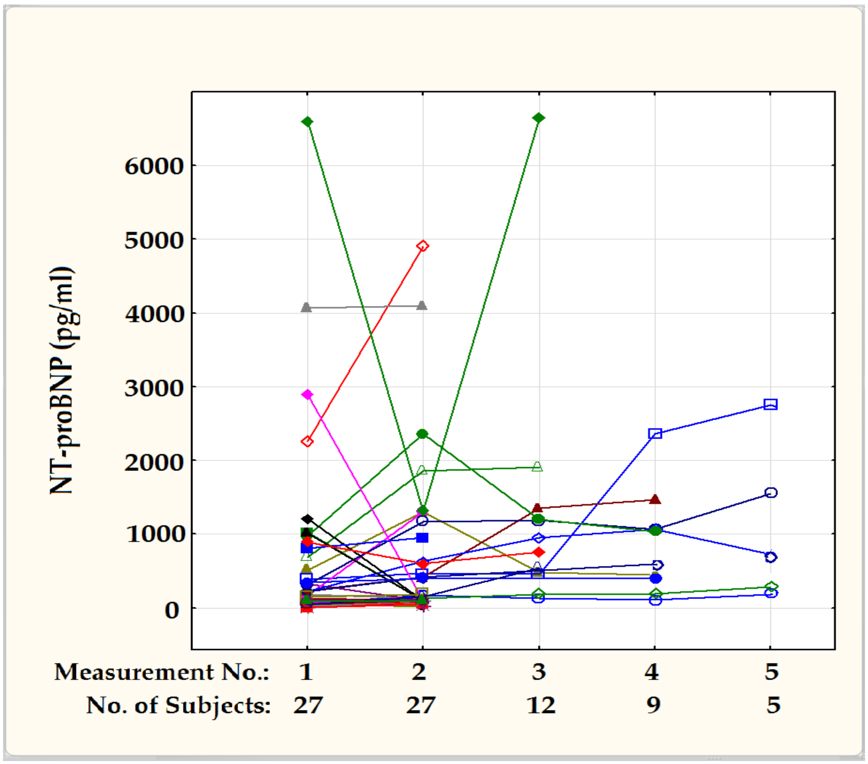

Figure S3. N-terminal pro-brain natriuretic peptide level profiles at baseline and during follow-up visits.

Table S4. Baseline clinical characteristics of *LMNA* variant carriers at initial visit according to mutation type.

	Non-Missens *n* = 40	Missense *n* = 13	*p*
Age (years)	34.0 ± 13.2	30.7 ± 9.5	0.405
Symptoms			
Syncope, *n* (%) (*n* = 49)	8 (21.6%)	4 (33.3%)	0.454
Family history of SCD < 60 years, *n* (%) (*n* = 50)	20 (51.3%)	5 (45.4%)	0.733
Heart failure, *n* (%)	12 (30.0%)	7 (53.8%)	0.183
NYHA class ≥ 3, *n* (%)	4 (10.0%)	3 (23.1%)	0.343
Arrhythmias			
Atrial arrhythmias, *n* (%) (*n* = 52)	16 (41.0%)	3 (23.1%)	0.328
nsVT, *n* (%) (*n* = 50)	22 (56.4%)	8 (72.7%)	0.489
SCA/sVT, *n* (%) (*n* = 50)	5 (13.2%)	4 (33.3%)	0.191
CCD			
LBBB, *n* (%) (*n* = 47)	4 (11.1%)	4 (36.4%)	0.073
AV block (≥ 1), *n* (%) (*n* = 52)	26 (65.0%)	5 (41.7%)	0.188
Cardiomyopathies			
LVEF < 50%, *n* (%)	11 (27.5%)	8 (61.5%)	**0.044**
LVEF (%)	53.7 ± 14.0	40.5 ± 19.1	**0.009**
LVE > 112%, *n* (%)	18 (45.0%)	10 (76.9%)	**0.045**
LVEDD (mm)	52.6 ± 7.7	57.2 ± 11.0	0.102
Biomarkers			
CK [IU/l] (*n* = 46)	176.0 (98–376)	85.0 (78–121)	**0.026**
elevated CK, *n* (%) (*n* = 46)	13/37 (35.1%)	0/9 (0%)	**0.044**
hs Troponin T (ng/L) (*n* = 42)	14.2 (9.9–25.4)	8.2 (5.0–14.6)	0.232
elevated hs Troponin T, *n* (%) (*n* = 42)	17 (51.5%)	3 (33.3%)	0.495

NT-proBNP (pg/mL) (n = 42)	129.5 (54–684)	225.0 (95–1029)	0.345
Elevated NT-proBNP, n (%) (n = 42)	16 (51.6%)	7 (63.6%)	0.726
Comorbidities			
Coronary artery disease, n (%)	2 (5.0%)	0 (0%)	1.000
Hypertension, n (%)	6 (15.0%)	0 (0%)	0.317
Implantable devices			
ICD in primary PPX, n (%)	4 (10.0%)	4 (30.8%)	0.090
ICD in secondary PPX, n (%)	5 (12.5%)	3 (23.1%)	0.389
ICD/CRT-D implantation, n (%)	9 (22.5%)	7 (53.8%)	**0.044**
Medication			
β-Blocker, n (%)	17 (42.5%)	8 (61.5%)	0.232
ACE-I or ARB, n (%)	14 (35.0%)	7 (53.8%)	0.227
MRA, n (%)	4 (10.0%)	2 (15.4%)	0.627

Legend: Number of subjects is expressed as n (%). Continuous variables are shown as mean ± SD or median and quartiles (Q1:25th–Q2:75th percentiles). ACE-I: angiotensin converting enzyme inhibitor; ARB: angiotensin receptor blocker; AV block: atrioventricular block; CCD: cardiac conduction defect; CK: creatine phoshokinase; CRT-D: cardiac resynchronization therapy defibrillator; hs: high sensitive; ICD: implantable cardioverter defibrillator; LBBB: left bundle branch block; LVE: left ventricular enlargement; LVEDD: left ventricular end-diastolic dimension; LVEF: left ventricular ejection fraction; MRA: mineralocorticoid receptor antagonist; nsVT: non-sustained ventricular tachycardia; NT-proBNP: N-terminal pro-brain natriuretic peptide; NYHA class: New York Heart Association functional class; PPX: prophylaxis; SCA: sudden cardiac arrest; SCD: sudden cardiac death; sVT: sustained ventricular tachycardia.

Table S5. Comparison of outcome according to diagnosis at initial visit.

**Outcome**	**DCM/HNDC** n **= 22**	**Indeterminate CM** n **= 21**	**No CM** n **= 10**	p
End-Stage HF	14 (63.6%)	0 (0%)	0 (0%)	**<0.0001**
MVA	9 (40.9%)	4 (19.0%)	0 (0%)	**0.034**
MVA + End-Stage HF	19 (86.4%)	4 (19.0%)	0 (0%)	**<0.0001**

Legend: CM: cardiomyopathy; DCM: dilated cardiomyopathy; HF: heart failure; HNDC: hypokinetic non-dilated cardiomyopathy; MVA: malignant ventricular arrhythmia.

Table S6. Clinical characteristics of *LMNA* variant carriers at last follow-up according to proband status.

	**Probands** n **= 21**	**Relatives** n **= 32**	p
Age (years)	44.2 ± 10.3	34.9 ± 12.6	**0.007**
Symptoms			
Heart failure, n (%)	19 (90.5%)	8 (25.0%)	**<0.0001**
NYHA class ≥ 3, n (%)	14 (66.7%)	3 (9.4%)	**<0.0001**
Arrhythmias			
Atrial arrhythmias, n (%) (n = 52)	14 (66.7%)	10 (32.3%)	**0.015**
nsVT, n (%) (n = 51)	19 (90.5%)	16 (50.0%)	**0.002**
CCD			
LBBB, n (%) (n = 39)	9 (64.3%)	1 (4.0%)	**<0.0001**
AV block (≥ 1°), n (%)	19 (90.5%)	17 (53.1%)	**0.004**
Cardiomyopathies			
LVEF < 50%, n (%)	18 (85.7%)	4 (12.5%)	**<0.0001**

LVEF (%)	32.7 ± 14.9	57.0 ± 9.5	**<0.0001**
LVE > 112%, *n* (%)	18 (85.7%)	11 (34.4%)	**0.0002**
LVEDD (mm)	61.3 ± 9.5	48.4 ± 10.0	**<0.0001**
Biomarkers			
hs Troponin T (ng/L) (*n* = 42)	21.4 (13.9–34.7)	13.2 (7.1–28.5)	**0.046**
elevated hs Troponin T, *n* (%) (*n* = 42)	11 (73.3%)	13 (46.4%)	0.090
NT-proBNP (pg/mL) (*n* = 41)	1211 (542–2245)	89.6 (51.5–422.0)	**<0.0001**
elevated NT-proBNP, *n* (%) (*n* = 41)	16 (94.2%)	10 (41.7%)	**0.0006**
Implantable devices			
ICD in primary PPX, *n* (%)	13 (61.9%)	13 (40.6%)	0.130
ICD in secondary PPX, *n* (%)	6 (28.6%)	2 (6.3%)	0.047
ICD/CRT-D implantation, *n* (%)	19 (90.5%)	15 (46.9%)	**0.001**
Events during follow-up			
Malignant ventricular arrhythmia, *n* (%)	7 (33.3%)	6 (18.7%)	0.227
Appropriate ICD shock, *n* (%)	7 (36.8%)	4 (12.5%)	0.075
RF ablation for VA, *n* (%)	5 (23.8%)	2 (6.2%)	0.099
Cardiopulmonary resuscitation, *n* (%)	0 (0%)	1 (3.1%)	0.413
Sudden cardiac death, *n* (%)	0 (0%)	1 (3.1%)	0.413
End-stage heart failure, *n* (%)	12 (57.1%)	2 (6.2%)	**<0.0001**
Heart transplantation, *n* (%)	9 (42.0%)	2 (6.2%)	**0.004**
HF death, *n* (%)	3 (14.3%)	0 (0%)	0.057

Legend: Number of subjects is expressed as *n* (%). Continuous variables are shown as mean ± SD or median and quartiles (Q1:25th–Q2:75th percentiles). AV block: atrioventricular block; CCD: cardiac conduction defect; CK: creatine phoshokinase; CRT-D: cardiac resynchronization therapy defibrillator; hs: high sensitive; ICD: implantable cardioverter defibrillator; LBBB: left bundle branch block; LVE: left ventricular enlargement; LVEDD: left ventricular end-diastolic dimension; LVEF: left ventricular ejection fraction; nsVT: non-sustained ventricular tachycardia; NT-proBNP: N-terminal pro-brain natriuretic peptide; NYHA class: New York Heart Association functional class; PPX: prophylaxis; RF ablation: radiofrequency ablation; SCA: sudden cardiac arrest; SCD: sudden cardiac death; sVT: sustained ventricular tachycardia; VA: ventricular arrhythmia.

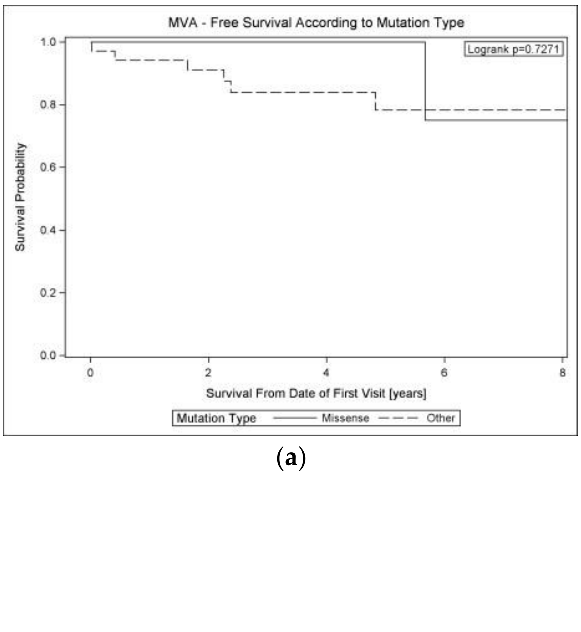

(**a**)

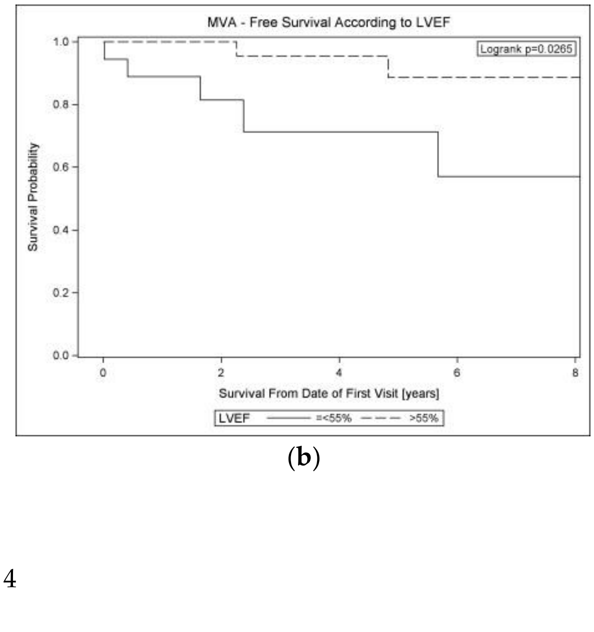

(**b**)

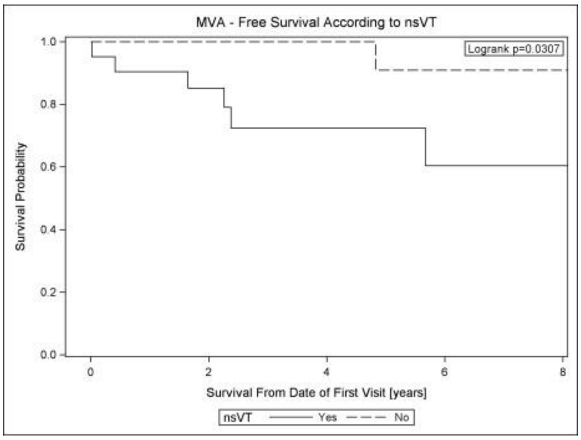

(**c**)

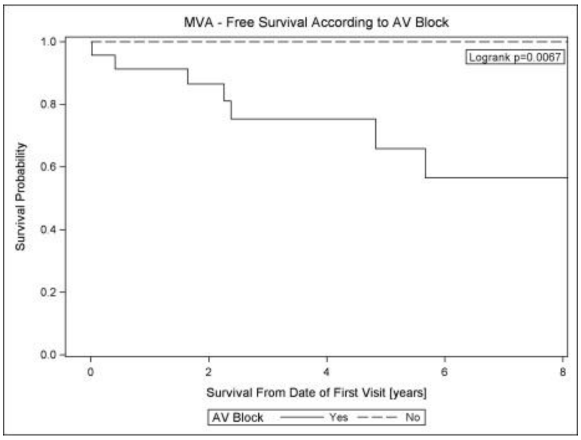

(**d**)

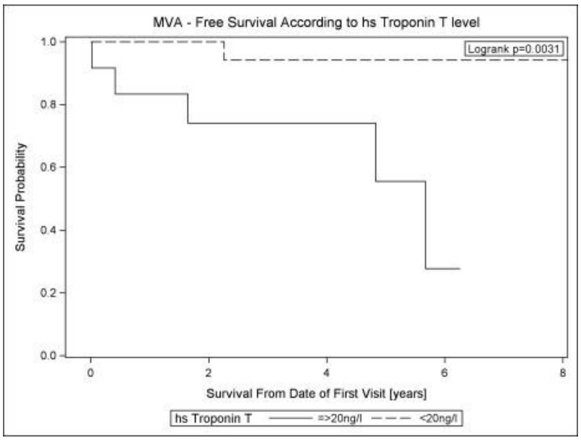

(**e**)

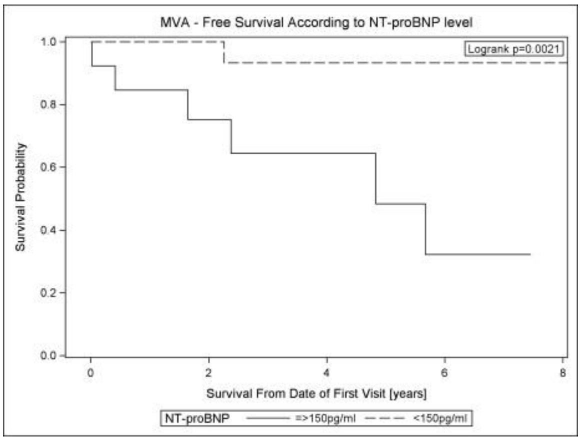

(**f**)

Figure S7. Kaplan–Meier MVA-free survival curves during follow-up in patients with cardiolaminopathy and no prior history of sudden cardiac arrest or sustained ventricular tachycardia according to (**a**) mutation type; (**b**) LVEF; (**c**) nsVT; (**d**) AV block; (**e**) Hs troponin T level; and (**f**) NT-proBNP Level. Legend: AV block: atrioventricular block; hs: highly sensitive; LVEF: left ventricular ejection fraction; MVA: malignant ventricular arrhythmia; nsVT: nonsustained ventricular tachycardia; NT-proBNP: N-terminal prohormone brain natriuretic peptide.

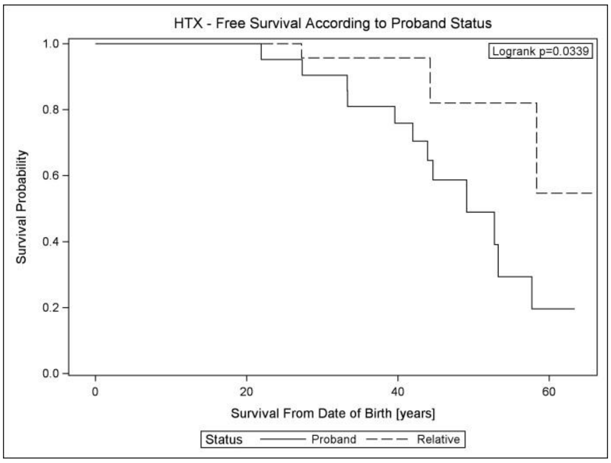

Figure S8. Kaplan–Meier lifelong HTX-free survival curves in cardiolaminopathy according to proband status. Legend: HTX: heart transplantation.

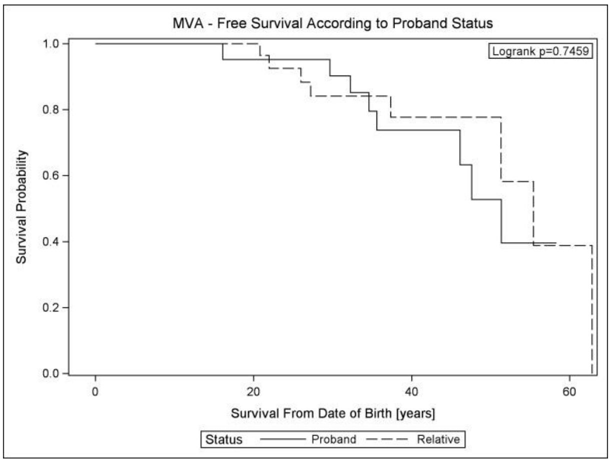

Figure S9. Kaplan–Meier lifelong MVA-free survival curves in cardiolaminopathy according to proband status. Legend: MVA: malignant ventricular arrhythmia.
